# Personalized medicine in triple-negative breast cancer: combining neoantigen vaccination and genomic profiling in a patient undergoing neoadjuvant chemotherapy

**DOI:** 10.3389/fonc.2025.1623402

**Published:** 2026-03-11

**Authors:** Laura Camila Martinez-Enriquez, David A. Bernal-Estévez, Daniel Alzate, Diego Amaya, Óscar Iván Reyes-Cortés, Carlos A. Parra-López

**Affiliations:** 1 Immunology and Translational Medicine Research Group, Department of Microbiology, Medical School, Universidad Nacional de Colombia, Bogotá, Colombia; 2 Immunology and Clinical Oncology Research Group, Fundación Salud de Los Andes, Bogotá, Colombia; 3 Deparment of Oncology, Hospital Universitario Nacional, Universidad Nacional de Colombia, Corporación UN, Bogotá, Colombia

**Keywords:** tumor neoantigen, dendritic cell vaccination, genomic profiling, triplenegative breast cancer (tnbc), neoadjuvant chemotherapy (NAC)

## Abstract

**Introduction:**

Triple-negative breast cancer (TNBC) is an aggressive subtype of breast cancer with the poorest prognosis among all breast cancer subtypes. The limited effectiveness of first-line treatments, such as systemic neoadjuvant chemotherapy (NAC) and surgery, underscores the urgent need for alternative therapeutic strategies. Next-generation sequencing (NGS) has revolutionized precision oncology by enabling the identification of tumor-specific neoantigens for personalized vaccines and the profiling of actionable mutations for targeted treatments. However, the application of these approaches in TNBC, particularly in the context of NAC, remains largely unexplored.

**Methods:**

We present a case study of a TNBC patient treated with NAC combined with dendritic cells (DCs) pulsed with a personalized tumor neoantigen vaccine. Whole-exome sequencing (WES) of germline and tumor tissue, along with tumor RNA sequencing (RNA-Seq), was performed to identify potential neoantigens. Candidate neoantigens were selected for vaccine development and evaluated for immunogenicity in peripheral blood mononuclear cells (PBMCs) through IFN-γ secretion assays, flow cytometric analysis of activation markers (CD69 and CD154) on CD4+ and CD8+ T cells, and T-cell receptor (TCR) CDR3 repertoire profiling after peptide stimulation. Germline and somatic variants were analyzed to assess hereditary risk and actionable mutations, respectively.

**Results:**

Selected neoantigens elicited IFN-γ responses in PBMCs prior to vaccination, indicating pre-existing immunity. Post-vaccination, enhanced immunogenicity was demonstrated by increased IFN-γ secretion, upregulation of activation markers CD69 and CD154 on CD8+ and CD4+ T cells, and polyclonal expansion of TCR CDR3 clones. Notably, an immune response against a non-vaccine neoantigen was observed following immunization, suggesting possible epitope spreading; however, responses detected in pre-vaccination samples preclude attributing this effect exclusively to vaccination. Germline WES identified a splice-altering pathogenic variant in *ATM* (c.2250G>A, p.Lys750=), confirming hereditary cancer predisposition. Somatic mutation analysis revealed alterations in *TP53* (p.Gly199Ter), NF1 (p.Phe1593SerfsTer31), and *PIK3CA* (p.Glu542Lys), all supported by OncoKB level-1 evidence.

**Discussion:**

This case highlights the potential of integrating precision oncology for TNBC patients undergoing NAC by identifying inherited cancer risks, detecting actionable mutations, and developing personalized neoantigen-based immunotherapies. The observation of immune responses to both vaccine and non-vaccine neoantigens raises the possibility of epitope spreading, though this finding requires cautious interpretation due to pre-existing immune responses. As a single-patient case study, these results remain preliminary and must be validated in larger cohorts.

## Introduction

According to the World Health Organization (WHO), breast cancer has the highest incidence worldwide and ranks fifth in cancer-related deaths (1, 2). In 2022, more than 2.3 million new cases were reported, with women experiencing the highest annual incidence—accounting for 23.8% of cases—and a mortality rate of 15.4% (3).

Breast cancer is a highly diverse disease classified into various subtypes. Triple-negative breast cancer (TNBC) is a subtype characterized by cancer cells that do not express receptors for estrogen (ER-), progesterone (PR-), and do not overexpress the HER2/neu (HER2-) oncogene (4, 5). TNBC makes up about 15% of all breast cancers and is associated with poorer survival rates and an early recurrence peak around three years after diagnosis and treatment. This tumor type exhibits high genetic instability, leading to significant resistance to chemotherapy (6, 7). As a result, TNBC displays aggressive clinical behavior, and most cases have low survival rates after five years, despite aggressive systemic chemotherapy and surgery (6). The high morbidity and mortality rates linked to this type of tumor are mainly due to the lack of effective treatment options. Consequently, current research focuses on discovering new therapeutic approaches.

The implementation of next-generation sequencing (NGS) has enabled the development of personalized medicine, revolutionizing cancer diagnosis and treatment by precisely identifying germline variants linked to hereditary predisposition and somatic mutations involved in tumor progression (8, 9).

The use of NGS to identify non-synonymous somatic mutations in the cancer cell exome has enabled the development of personalized vaccines based on neoantigens (10). These epitopes are believed to have strong immunogenic potential because the mutated sequences differ from their wild-type counterparts and should therefore be recognized by the immune system as foreign (11). As a result, they serve as ideal therapeutic targets because they can enhance the specificity of the immune response, overcome immunological tolerance, and potentially produce a long-lasting therapeutic effect. In patients with melanoma and metastatic lung cancer, neoantigens are presented in the context of MHC class I and class II molecules, allowing recognition by both CD8^+^ and CD4^+^ T cells, which can trigger a robust antitumor response (12–18).

The genetic instability of TNBC makes it an ideal target for designing personalized neoantigen-based vaccines due to its high mutation rate, which leads to a large number of neoantigens (19, 20). As a result, identifying nonsynonymous mutations and vaccinating with peptides that incorporate these mutations is emerging as a tool for cancer immunotherapy (21, 22), and these vaccines can be formulated as synthetic peptides (15, 16) or delivered using autologous dendritic cells (DCs) as a vehicle (12, 23).

Exome sequencing can also identify germline and somatic variants of clinical importance. Detecting germline mutations helps identify genes linked to cancer diagnosis and hereditary risk (24, 25). Conversely, finding somatic mutations is vital in precision medicine because it uncovers potential therapeutic targets and driver genes (26, 27). Therefore, accurately detecting these changes is crucial for precision medicine, as it influences the choice of targeted treatments and the management of genetic risks for patients and their families. In this regard, establishing standardized, reliable, and reproducible bioinformatics workflows is essential to manage the complexity and molecular diversity of cancer.

We present a case of a patient with triple-negative breast cancer who underwent whole-exome sequencing of both healthy and tumor tissue, as well as transcriptome analysis of the tumor (before and after NAC), to identify somatic and germline mutations. This approach enabled the identification of tumor-specific epitopes, which were used to vaccinate the patient with autologous DCs loaded with the corresponding neoantigen. The vaccination elicited an immune response against the neoantigen and boosted T cell responses to an additional neoantigen not included in the vaccine. Furthermore, the analysis of genetic variants revealed a mutation associated with hereditary cancer predisposition, along with mutations in oncogenes that may serve as actionable therapeutic targets.

## Methodology

### Study design

**Figure 1:**
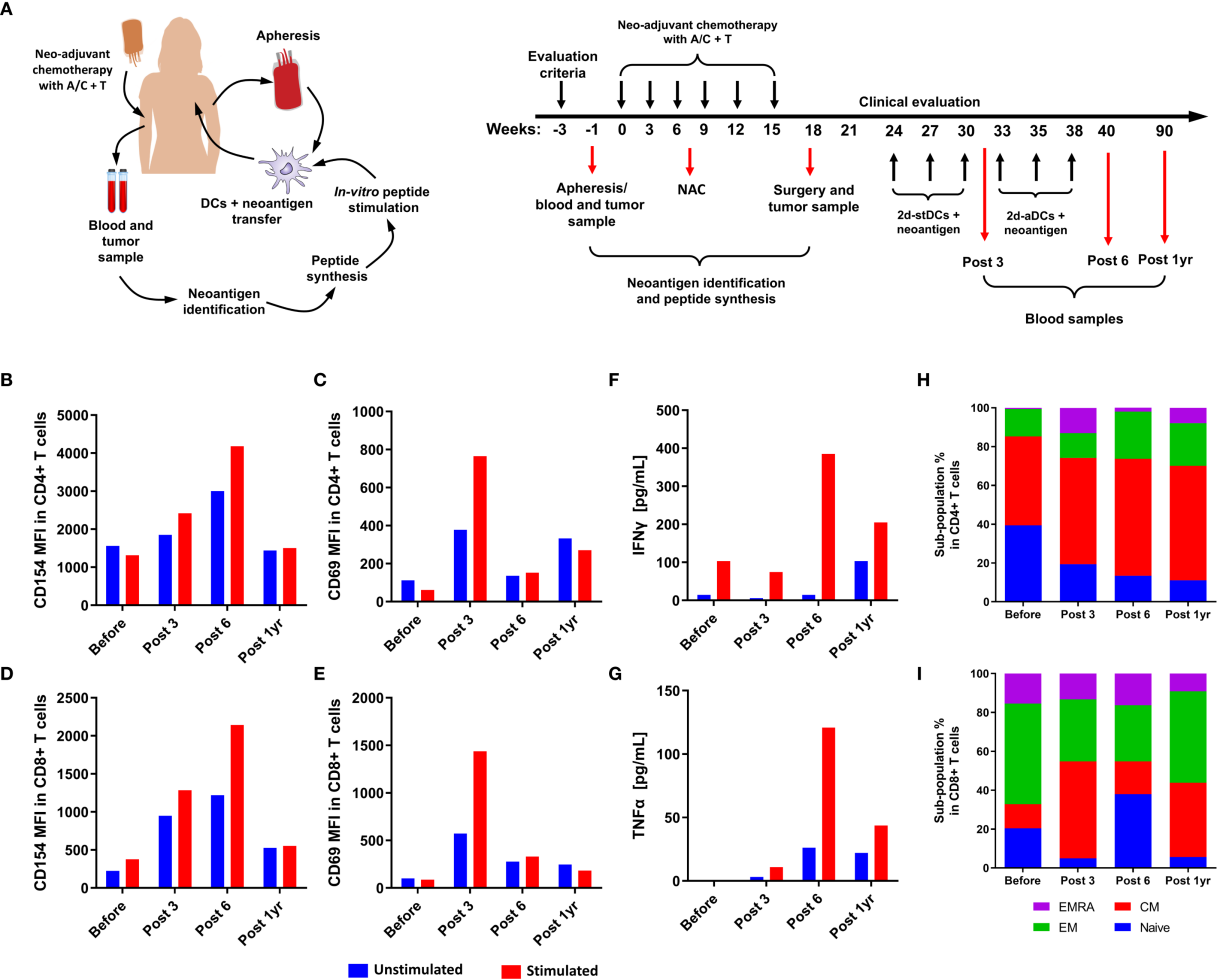
Evaluation of T-cell activation and cytokine secretion in response to neoantigen stimulation. Clinical trial scheme and sample collection **(A)**. Expression of the activation markers CD154 **(B, D)** and CD69 **(C, E)** in CD4 and CD8 cells of the patient’s PBMCs, as well as the secretion of cytokines IFN-γ and TNF-α **(F, G)** at different post-vaccination times: pre-vaccination, after the third dose, after the sixth dose, and one year post-vaccination. Distribution of memory phenotypes, determined by CD45RA and CCR7 markers, in CD4+ **(H)** and CD8+ **(I)** T cells of the patient’s PBMCs at various post-vaccination times: before, after the third dose (Post 3), after the sixth dose (Post 6), and one year after vaccination (Post 1yr). In blue: T-Naive (CD45RA+ CCR7+); in red: T-CM (CD45RA- CCR7+); in green: T-EM (CD45RA- CCR7-); in purple: T-EMRA (CD45RA+ CCR7-).

This clinical study (NCT04105582) received approval from the ethics committee of Universidad del Rosario and Hospital Mayor de Méderi (Act. No. 409, February 27, 2020) in Bogotá, Colombia. Patients with a clinical diagnosis of triple-negative breast carcinoma, who provided informed consent and signed the consent form, were enrolled in the study. A blood sample was collected via apheresis for immunogenicity analysis and for generating autologous DCs (see methodology below). Tumor tissue was obtained through Tru-Cut biopsy before starting neoadjuvant chemotherapy to identify tumor neoantigens. Blood samples were also collected during treatment, at 6 months, and one year after the last dose of autologous DCs pulsed with the neoantigens (**Figure 1A**).

The FMR patient who participated in the clinical study is a 53-year-old female with a diagnosis of moderately differentiated nonspecific ductal NOS infiltrating carcinoma, grade II/II, triple-negative, Ki67 30%, T2 N1 M0, stage IIB. Mammography in 2019 revealed a mass in the external upper quadrant of the left breast measuring 30x22x29 mm, with an associated lymph node of 22x13 mm. The patient underwent a hysterectomy at age 39. Her family history includes her father as the only relative with gastric cancer. She received an AC-T regimen (4 doses), followed by a left quadrantectomy with lymph node dissection (one of 13 nodes was positive). After surgery, she completed 20 sessions of radiotherapy. Currently, she is being treated with tamoxifen, with no signs of local or regional recurrence.

### Prediction of the patient’s tumor neoantigens

An integrated bioinformatics workflow was developed for processing exome and transcriptome sequencing data from paired normal and tumor samples. Initially, reads were aligned to the GRCh38 reference genome using HISAT2 with parameters optimized for assembly compatibility. The resulting SAM files were converted to BAM format, sorted, and filtered to remove unmapped reads and duplicates using SAMtools and Picard. Somatic variants (SNVs and indels) were identified with GATK MuTect2 by comparing the tumor exome to its corresponding normal control (peripheral blood exome). A filtering process was then applied with GATK FilterMutectCalls to ensure high-quality variant calls. Variants were normalized (bcftools norm -m-both), decomposed (vt decompose), and compressed (bgzip), with index files generated using tabix. Functional annotation was performed using VEP (with plugins: Frameshift, Wildtype; options: –cache, –hgvs, –vcf), incorporating gene expression data from Cufflinks (FPKM) and StringTie. Read counts from bam-readcount and transcriptional/gene expression data from vcf-expression-annotator were annotated in a stratified manner for exome and transcriptome, using a specific threshold (-b 20 for base quality).

HLA typing data were obtained using OptiType (28) and seq2HLA (29) from the patient’s normal exome and transcriptome, respectively, while neoantigen prediction was performed with the pVAC-seq suite (30). This suite predicts mutated high-affinity peptides derived from identified somatic mutations. Neoepitope prediction was conducted for the tumor sample using the patient’s HLA genotype, which included both class I and class II alleles. Multiple predictive algorithms (NetMHCpan, NetMHC, and NetMHCIIpan) were used, considering peptides of 8–11 amino acids for HLA class I and 12–25 amino acids for HLA class II. Additional protein processing (NetChop) and stability (NetMHCstab) methods were employed to evaluate proteasomal cleavage characteristics and the binding stability (half-life) of the peptide/MHC complexes (NetMHCstab predicts only with HLA class I molecules). Neoantigen prioritization was based on binding affinity (IC50 < 500 nM across multiple algorithms), mutated transcript expression (FPKM > 1), and NetMHCstab half-life stability time (greater than 1 hour). Five class I neoantigens and one class II neoantigen were identified. Due to budget limitations in peptide synthesis capabilities, the class II peptide was selected, which also contained one of the identified class I epitopes. Detailed features of all five class I and the single class II neoantigens are provided in **Supplementary Table 1**.

### Vaccine generation and administration

The generation of autologous DCs was based on a previous study (31). Briefly, enriched monocytes were obtained from an apheresis sample of the patient. Isolated peripheral blood mononuclear cells (PBMCs) were stored in liquid nitrogen until use for DCs generation for each dose. During neoadjuvant chemotherapy, tumor and blood samples were used to extract DNA and RNA for neoantigen identification, as described above for synthesis (one peptide per patient). A week before each dose, frozen PBMCs were thawed, and enriched monocytes were then induced into immature DCs with the addition of IL-4 and GM-CSF and finally matured with two cocktails of pro-inflammatory cytokines. During maturation, the cells were stimulated with the corresponding neoantigen (20μg/mL), defined from each patient’s specific tumor. After several washes with saline solution, a total of 1.2x10^8 autologous DCs stimulated with the neoantigen were divided in six doses after neoadjuvant chemotherapy, intradermally in the draining region of the tumor (**Figure 1A**). Patients were monitored for 4 hours after vaccination to detect any adverse effects, then clinical follow-up was carried out every 24 hours until the next dose. Finally, follow-up continued monthly after the last dose until month 12.

### Vaccine immunogenicity evaluation

#### Culture system

PBMCs were collected before immunization and after the final dose of vaccination. These cells were isolated using Ficoll’s density gradient and counted with trypan blue to assess viability before cryopreservation. We adapted a methodology previously developed by Martinuzzi et al. (32) with some modifications. Briefly, frozen-thawed PBMCs were seeded at the following densities in AIM-V (Thermo Fisher Scientific) medium: 1 x 10^6 cells per 96-well round-bottom plate. The cytokines used for accelerated culture DC stimulation were added sequentially: for day 0, GM-CSF (R&D Systems; 1,000 U/mL), IL-4 (R&D Systems; 500 U/mL); for day 1, LPS (10 ng/mL, Invitrogen; 0.5 mg/mL) and IFN-y (R&D Systems; 1,000 U/mL); and for day 2, IL-2 (Proleukin, Novartis; 100 U/mL). These cytokines were added by replacing half of the medium volume with AIM-V containing the respective cytokines at the final concentrations calculated for the entire culture volume. Half of the medium was replaced every 2–3 days with AIM-V supplemented with 100 U/mL of IL-2. After 24 hours of peptide restimulation, the supernatant was frozen for cytokine measurement, and cells were collected for flow cytometry analysis.

#### Characterization of CD4+ and CD8+ T cell responses

After 24 hours, cells were collected and washed once with phosphate-buffered saline (PBS) (Sigma) and stained with monoclonal antibodies to CD3 in Pacific Blue (Biolegend), CD4 Brilliant Violet 510, CD8 PE-Dazzle 594, CD45RA APC, CCR7 FITC, CD69 APC-Cy7, 4-1BB PE-Cy5, and PD-1 PerCP-Cy5.5. Following 20 minutes at 4°C in the dark, the cells were fixed and permeabilized using the Intra stain (Dako) kit, then labeled with CD154 PE and CTLA-4 PE-Cy7 for intracellular staining. Samples were acquired with the FACSAria III System from the National University of Colombia. Flow cytometry data were exported in FCS v3 format and analyzed with FlowJo software (BD) and R for unsupervised analyses. The graphs were generated using Prism v6 software (GraphPad).

#### IFN-y ELISPOT Assay

Autologous PBMCs were used as APCs prior to vaccination. These cells were pulsed overnight with the indicated peptides at a final concentration of 10 µg/mL at 37°C. PBMCs from the expanded culture were seeded in plates at a concentration of 2-3x10^5 cells per well in 200 μL of serum-free C.T.L. (Cellular Technology, Ltd.) on pre-coated and peptide-stimulated (10 μg/mL) human IFNγ ELISPOT plates. A Cell Stimulation Cocktail (PMA/Ionomycin, Biolegend) was used as a positive control at 1X concentration. The plates were incubated for 48 hours at 37 °C and analyzed using *ImageJ.*

#### Measurement of cytokines in supernatant by CBA

The supernatants from different cultures and donors were thawed and diluted 1:1 in PBS before incubation with CBA beads coated with anti-IL-2, IL-6, TNF-α, and IFNγ antibodies (CBA Th1/Th2 II kit; BD Biosciences). The beads were washed and stained with the PE-conjugated secondary antibody. The concentrations of each cytokine in the supernatants were analyzed using the FCAP array v3 software (BD Biosciences).

#### TCR sequencing and analysis

Those samples were immediately processed for single-cell suspension and cryopreserved as described earlier. DNA was extracted from PBMCs before chemotherapy and vaccination (Before), after three cycles of chemotherapy (NAC), and following the 3rd (Post 3) and 6th (Post 6) doses using the DNeasy^®^ Tissue and Blood DNA Extraction Kit (Qiagen, Germany), following the manufacturer’s protocol. The TCR-Vβ CDR3 regions were then sequenced using ImmunoSEQ technology (Adaptive Biotechnologies, Seattle, WA, USA). Frequencies of TCRB in each sample were analyzed using the ImmunoSEQ platform to generate 2D dot plots, Venn diagrams, and the productive frequency of each TCRB.

We used the patient’s tumor RNA data and the TRUST4 (33) algorithm to deconvolute and identify TCRs (specifically CDR3 regions) of the lymphocytes infiltrating the tumor in both pre- and post-chemotherapy samples. We compared these results with the sequences of the clonotypes we identified using ImmunoSEQ. This enabled us to identify CDR3s shared between the peripheral blood and the tumor-infiltrating lymphocytes.

#### Measurement of intracellular cytokines by flow cytometry

Autologous PBMCs pulsed with the peptide(s) were used as presenting cells in the flow cytometry intracellular cytokine staining assay. To do this, the PBMCs were thawed and pulsed for 16 hours with the different peptides or peptide pools at a final concentration of 10 μg/mL per peptide at 37°C. These cells were then labeled with succinimidyl-carboxyfluorescein ester (CFSE; Biolegend) and co-cultured with the cultured cells at a 1:1 ratio, with a density of 2×10^5 cells in 96-well round-bottom plates. To evaluate the specificity of the response, the negative control was the co-culture of the cultured cells with autologous PBMCs without peptide, while an activation cocktail was used as a positive control (25 ng/mL of PMA and 1 μg/mL of Ionomycin; Biolegend). After 1.5 hours of co-culture, 5 μg/mL of Brefeldin A (Biolegend) was added to the culture medium, and incubation continued at 37°C until 6 hours of stimulation were completed. After this incubation, cells were washed with PBS and stained with the viability marker (Aqua, Thermo). Surface labeling was performed for 20 minutes with the following antibodies: anti-CD8 (PE/Dazzle594, Biolegend) and anti-CD3 (Pacific Blue, Biolegend). The cells were fixed and permeabilized using the Intrastain kit (DAKO), and intracellular labeling was done with anti-TNF-α (APC, Biolegend) and anti-IFNγ (PE, Biolegend) antibodies for 20 minutes in the dark. Cells were then washed with 1 mL of PBS for 7 minutes at 700xg, resuspended in 200 μL of PBS, and at least 5×10^4 events were acquired per experiment on a FACSAria IIIu cytometer. Data were analyzed in FlowJo software to determine the populations of CD8+ T cells producing IFNγ and TNF-α within the Aqua- CFSE- cell population.

### Germline variant detection based on GATK best practices

The pipeline for germline variant detection was developed following GATK Best Practices (34) to ensure a robust and reproducible process. First, read quality is evaluated with FastQC (35), and reads are aligned to the reference genome (hg38) using BWA-MEM (36). The resulting files (SAM/BAM) are converted, sorted, and indexed with Samtools. Next, variant calling with GATK is optimized, including duplicate marking (MarkDuplicatesSpark), alignment sorting (SortSam), and base quality recalibration (BaseRecalibrator and ApplyBQSR). Germline variants are called with HaplotypeCaller (37). Finally, functional annotation is performed using Ensembl VEP and the Franklin Genoox platform, integrating information on allele frequency, molecular effects, and clinical annotations. The validity of the pipeline was confirmed by achieving 100% agreement in variant detection in a set of independent samples previously analyzed in a clinical setting (Euformatics Genomics Hub).

### Consolidation of a cancer driver gene panel

To prioritize germline variants potentially affecting cancer predisposition, they were selected from a panel of driver genes compiled from the literature (38–41) and gene panels used in clinical diagnosis (Illumina TruSight Oncology, Pan-cancer panel CD Genomics). Within the IntOGen database, genes present in at least 1% of cancer patients were chosen. From the OncoKB database, genes confirmed as cancer drivers in at least three sources were selected, citing the gene as a cancer driver.

### High-confidence somatic variant detection

For the identification and annotation of somatic variants in tumor exomes, a stepwise bioinformatics pipeline was designed. The raw reads were first evaluated with FastQC (35). They were then aligned to the hg38 genome using BWA-MEM (36). The generated files were processed with GATK to remove duplicates (MarkDuplicatesSpark) and recalibrate base quality scores (BaseRecalibrator and ApplyBQSR) (42). Subsequently, somatic variant calls were made in parallel using MuTect2 (43), LoFreq (44), MuSE2 (45), and Strelka2 (46). The results from the different somatic callers were integrated with SomaticSeq (47), which employs machine learning to prioritize the most reliable variants. Finally, the variants were annotated with Ensembl VEP (48) and converted to MAF format using vcf2maf to incorporate them into Memorial Sloan Kettering’s OnkoKB oncology database (41), enabling the identification of mutations with clinical significance based on each patient’s tumor genomic profile (49). The entire pipeline was executed within Docker containers to ensure reproducibility of the analyses.

## Results

### Follow-up neoantigen immunogenicity before and after vaccination

Since the frequency of circulating cells specific to a tumor neoantigen is very low, an expansion protocol was developed in which the patient’s PBMCs were stimulated with cytokines to induce the differentiation and maturation of DCs in the presence of the neoantigen. We used one approach that links antigen processing to the presentation of the neoantigen to specific T cells by DCs *in situ*, published by Martinuzzi et al. (32), with minor modifications. The PBMCs were pulsed on day 0 and re-stimulated on day 9 with the neoantigen to evaluate cytokine secretion and the expression of activation markers on T cells, as assessed by flow cytometry, which serve as indicators of neoantigen recognition. This assay was conducted with PBMCs at multiple time points: before, post 3, post 6, and one-year post-vaccination (Post 1 yr).

As shown in **Figure 1**, re-stimulated cells exhibited increased expression of CD154 and CD69 activation markers in both CD4+ and CD8+ T cells compared with the non-re-stimulated control. CD154 expression was sustained across time points, peaking after the sixth dose (Post 6) and declining one year after immunization (**Figures 1B, D**). In contrast, CD69 expression was most pronounced at the third dose (Post 3) and decreased at subsequent time points (**Figures 1C, E**). Cytokine secretion reflected these activation marker profiles: both IFNγ and TNF-α increased progressively over time, reaching a peak after the sixth dose (Post 6), and then declined by the one-year follow-up (Post 1yr) (**Figures 1F, G**). Altogether, these results demonstrate that vaccination induced a measurable neoantigen-specific T-cell response characterized by activation marker expression and cytokine secretion, which peaked at Post 6 but waned one year after vaccination.

### Change in the T cell memory phenotype induced by neoantigen vaccination

To determine if vaccination altered the memory phenotype, the patient’s PBMCs were stimulated using the same culture system described earlier, with CD45RA and CCR7 markers used to evaluate the memory phenotype. Different behaviors were observed in CD4+ and CD8+ T cells. For CD4+ T cells, data over time showed a decrease in naive T cells and an increase in central memory cells when stimulated with the neoantigen (**Figure 1H**). For CD8+ T cells, the effector phenotype is dominant before vaccination; however, as the vaccination schedule continues, the percentage of central memory cells increases. After the sixth dose (Post 6), the naive phenotype becomes predominant, but at one year, the naive population decreases while central memory and effector memory cells increase (**Figure 1I**). These findings indicate a shift in the memory phenotype of both CD4+ and CD8+ T cells, more noticeably in CD4+ T cells, which show an increase in the central memory phenotype. In contrast, no clear pattern was observed for CD8+ T cells.

### Changes in peripheral T-Cell Receptor repertoire after NAC and vaccination

**Figure 2:**
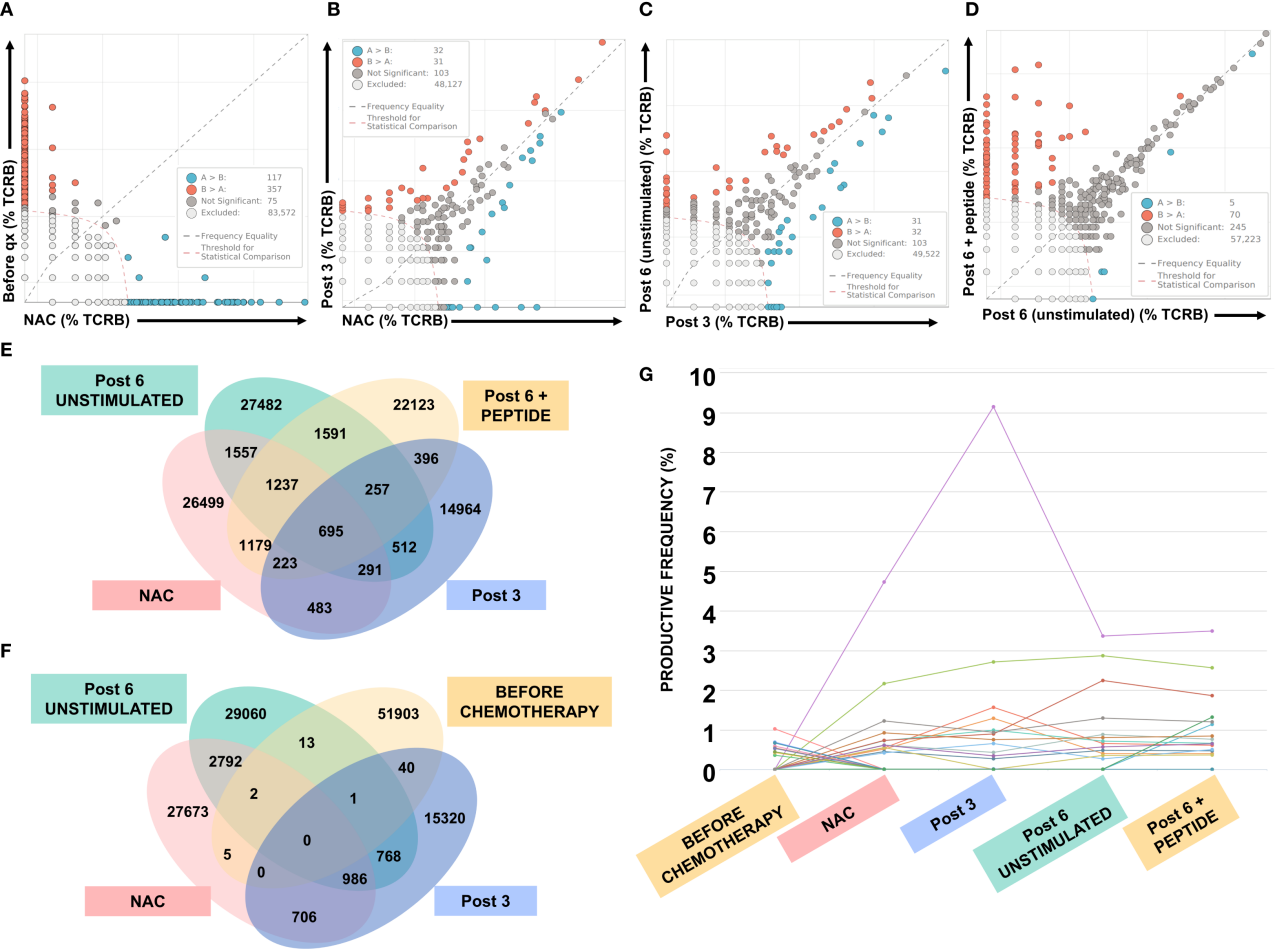
T cell repertoire analysis by CDR3 sequence. Differential expansion of TCR-CDR3 regions before chemotherapy, after the third dose of NAC (Qx), and after the third and sixth doses of DC pulsed with neoantigen (unstimulated and after *in vitro* stimulation). **(A-D)** Plots of TCRB frequency across five samples: orange and blue dots show significantly increased frequency in the respective sample (y and x axes, respectively), dark gray dots indicate TCRB shared between samples, and light gray represents low-frequency TCRB. **(E, F)** Venn diagrams display the number of common and unique TCRB among samples. **(G)** Productive frequency of the most significant TCRB shown as a continuum in each sample.

To evaluate the immune response related to changes in the T cell repertoire at different vaccination times, we measured the frequency and sequence of the TCR-CDR3 region of T cells in PBMCs collected at various time points: before chemotherapy and vaccination (Before), after three cycles of chemotherapy (NAC), and Post 3 and Post 6 doses of the vaccine. Additionally, a comparison between unstimulated and neoantigen-stimulated T cells was performed at the Post 6 dose time point. To identify significant differences in CDR3 frequency between samples, we plotted two samples on a scatter diagram to detect rearrangements (unique sequences) that significantly increased or decreased in frequency (**Figure 2**). Most sequences were excluded due to low frequency (**Figure 2A**); however, some rearrangements were significantly more common in the pre-treatment (Before) group (orange dots, n = 357), while others increased after chemotherapy (blue dots, n = 116) (Before qx Vs. NAC in **Figure 2A**). When comparing the rearrangements of NAC samples versus Post 3 (**Figure 2B**, NAC Vs. Post 3), and Post 3 versus unstimulated Post 6 (**Figure 2C**, Post 3 Vs. Post 6) we observed similar patterns, as many rearrangements are shared, with a few unique to each treatment. We used a Venn diagram to assess the number of overlapping sequences in each sample (**Figure 2E**). There are no common sequences among the four time points (Before, NAC, Post 3, and Post 6 unstimulated); however, many sequences are shared between the last two treatment time points (**Figure 2E**). These results suggest that, in this patient, the combined treatment of NAC and vaccination may promote the expansion of circulating T cells with identical TCR-CDR3 sequences.

### *In vitro* stimulation shows polyclonal expansion of neoantigen-responsive CDR3 clonotypes

Unlike the overall analysis of the TCR repertoire described above, the comparison between unstimulated and neoantigen-stimulated Post 6 cells showed that, although many sequences were shared, 70 rearrangements were more common in the stimulated condition (Post 6 + peptide vs. Post 6 unstimulated, orange dots in **Figure 2D**). Paired analyses of TCR-CDR3 sequences across different time points indicated that most rearrangements expanded after chemotherapy and the Post 3 compared to pre-chemotherapy (Before) samples, while some only increased when Post 6 cells were stimulated with the neoantigen (**Figure 2G**). The observed polyclonal expansion is attributed to the neoantigen, based on TCR sequencing results from stimulated versus unstimulated PBMCs, suggesting expanded clonotypes that likely recognize the neoantigen. However, this interpretation depends on antigen-specific stimulation. Overall, these findings demonstrate the usefulness of the expanded cultures in showing the vaccine’s immunogenicity and identifying candidate CDR3 clonotypes that may be specific to the neoantigen used in vaccination.

### Chemotherapy and/or neoantigen vaccination promotes the emergence of shared tumor–blood TCR clonotypes

CDR3 sequences were identified from RNA-seq performed on pre-treatment and post-chemotherapy/vaccination tumor biopsies, enabling comparison between tumor-derived clonotypes and those detected in peripheral blood. A higher number of shared CDR3 sequences was observed between the post-chemotherapy tumor and peripheral blood samples across all evaluated time points (Before, NAC, Post 3, and Post 6), suggesting an increased overlap of circulating and tumor-infiltrating T cells after therapy. No CDR3 sequences were shared between the pre-treatment peripheral blood sample (before) and either of the tumor samples. Interestingly, we identified one CDR3 sequence (CASSEEGLHNEQFF) present in both pre- and post-treatment tumors as well as in PBMCs from all post-treatment time points, but absent in pre-treatment PBMCs, indicating the emergence of a tumor-reactive clonotype in the circulation following chemotherapy. Additionally, analysis of PBMCs collected after the sixth vaccination dose (Post 6) and stimulated *in vitro* with the peptide revealed a clonotype shared with tumor samples; however, distinct clonotypes were observed when comparing pre-treatment (CSARKVASYNEQFF) and post-treatment (CASSPQGEEQFF) tumors, consistent with the induction of a vaccination-associated neoantigen-specific clonotype. The detailed results of these comparisons are provided in **Supplementary Table 2**.

### Immune responses to vaccine and non-vaccine neoantigens

To evaluate the immunogenicity of four identified neoantigens not included in the vaccine, we investigated whether vaccination combined with NAC promoted responses to these epitopes, a phenomenon referred to as “epitope spreading.” To this end, PBMCs collected after the sixth vaccination dose (Post 6) were subjected to an expansion protocol and stimulated with a pool of the four neoantigens, each derived from single amino acid mutations and exhibiting distinct HLA-I restriction (**Table 1**).

**Table 1:** Neoantigen characteristics and mutated epitopes identified.

Peptide Name	Gene	AA Change	MT Epitope	IC_50_ (nM)		DNA VAF (%)	RNA VAF (%)	HLA Restriction
				WT	MT			
Pep-1	OR2T33	V160A	LQAVATLSF	2.7	2	30.4	34.8	HLA-B*15:03
Pep-2	OR3A2	T42I	LLFAYLVTI	36.7	9.2	36.4	33.7	HLA-A*02:01
Pep-3	SIPA1L3	A378T	SAASATSAM	90.8	69	28.6	26.7	HLA-B*15:03
Pep-4	TRPM3	R367W	SGWASDILAF	360.1	420.8	31.9	32.7	HLA-B*15:03
Neoantigen Vaccine	AES	Class I epitope	Class II epitope	IC_50_ MT Class I (nM)	IC_50_ MT Class II (nM)	26.6	—	HLA-B*15:03
		HQLSQLQAL	QQLQAHQLSQLQA LALPLTPLPVGL	10	151			HLA-DPA1*02:01 - DPB1*107:01

**Figure 3:**
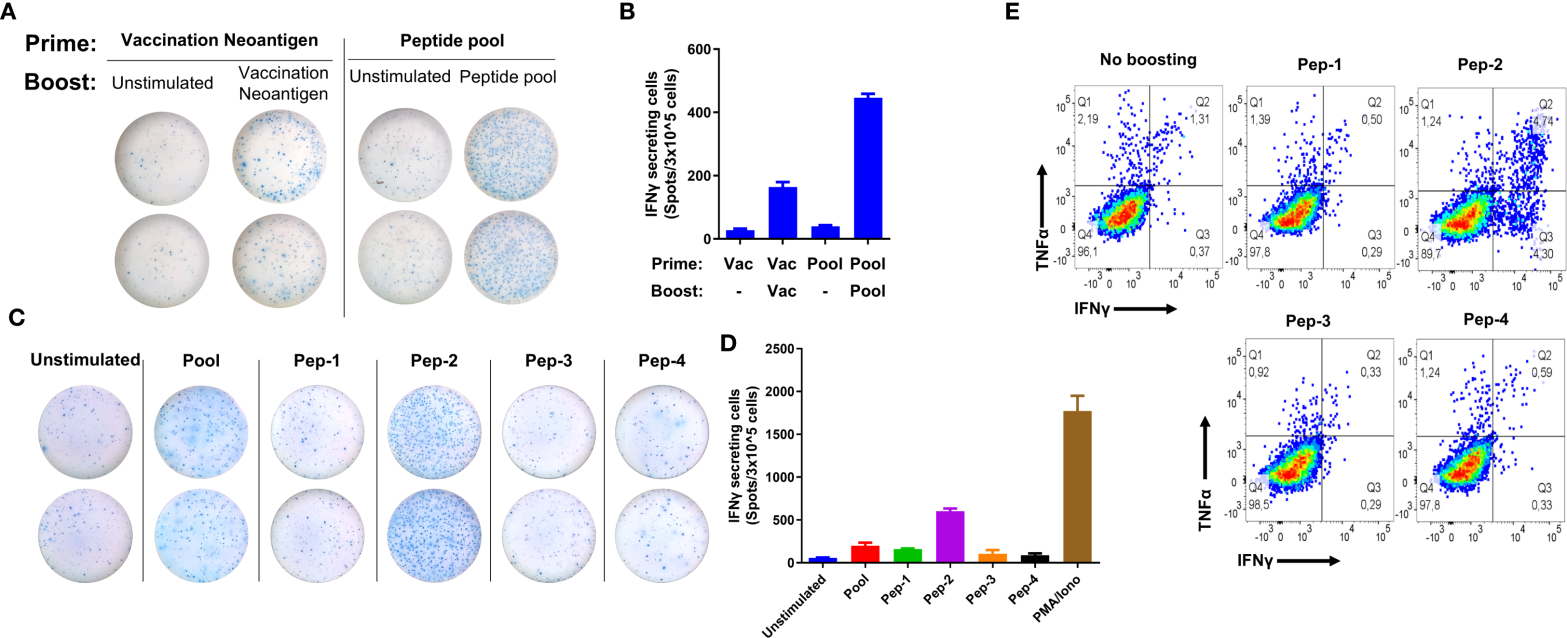
Evaluation of T cell response to predicted neoantigens not used for vaccination. ELISPOT wells display the response of IFN-γ-producing T cells after the sixth dose of vaccination. The cells were stimulated *in vitro* with both the vaccine neoantigen and a pool of four predicted neoantigens not included in the vaccine **(A)**. Quantification of spots **(B)**. Immune responses, as measured by the ELISPOT of IFN-γ in post-vaccination cells previously stimulated with a pool of neoantigens, were individually re-stimulated with each peptide **(C)**. The response was quantified by IFN-γ ELISPOT, showing a more significant response to Pep-2 **(D)**. Evaluation of intracellular expression of IFN-γ and TNF-α by flow cytometry before and after restimulation with individual peptides from the neoantigen pool **(E)**.

To assess the immune response to both the neoantigen used for vaccination and the pool of four neoantigens not included in the vaccine, an IFNγ ELISPOT assay was performed to quantify cytokine secretion as an indicator of T-cell activation. As previously reported, a response to the vaccine-derived peptide was confirmed. Additionally, a robust response to the neoantigen pool was observed, prompting further *in vitro* analyses to identify the specific peptide(s) driving IFNγ production (**Figures 3A, B**). To achieve this, the pool was deconvoluted by re-stimulating the cells from the stimulated pool with individual peptides. Cytokine expression was then evaluated using IFNγ ELISPOT and by measuring intracellular IFNγ and TNF-α through flow cytometry. Both assays demonstrated a clear cytokine secretion in response to peptide 2 (Pep-2) (**Figures 3C, D**), with 7.74% of cells co-expressing IFNγ and TNF-α (**Figure 3E**).

### Characterization of Pep-2–specific immune responses across treatment time points

**Figure 4:**
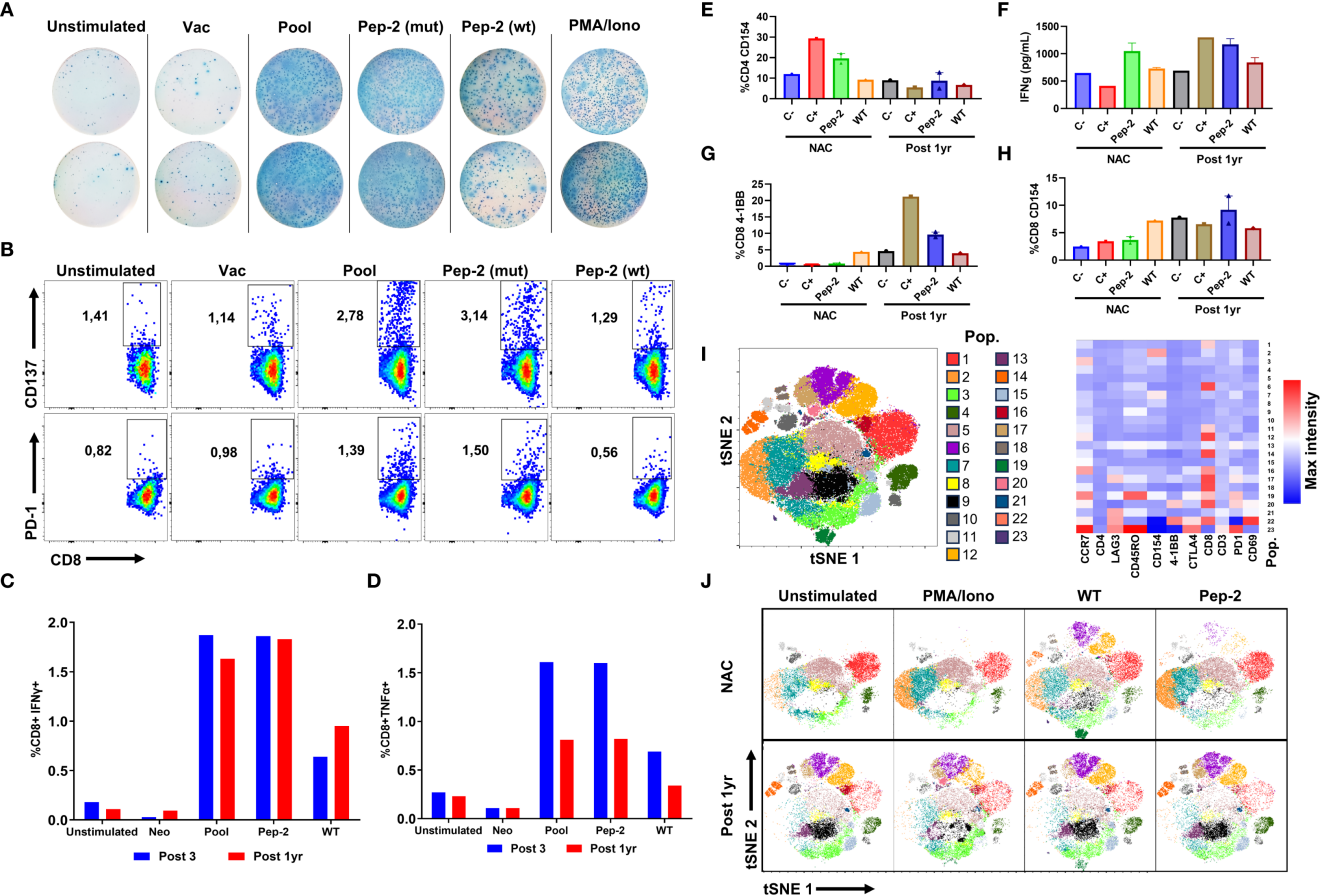
Characterization of the immune response to the identified neoantigen not included in vaccination. **(A)** Comparison of the immune response to the mutated peptide and its wild-type (WT) version in PBMCs one year after vaccination, assessed by IFN-γ ELISPOT. **(B)** CD137 and PD-1 activation markers measured by flow cytometry **(C)** and IFN-γ and TNF-α production by flow cytometry **(C, D)**. **(E)** Percentage of activated CD4+ CD154+ **(F)** IFN-γ secretion in PBMCs supernatant. **(G)** Expression of 4-1BB in CD8+ T cells, and CD154 expression in CD8+ T cells **(H)**. **(I)** Dot plot and heat map by tSNE of the concatenated samples with identification of 23 populations using Phenograph, illustrating the expression of different markers in each of the 23 populations. **(J)** tSNE plots with populations discriminated by Phenograph for each culture condition.

Once Pep-2 was identified as the main driver of the immune response, the response elicited by this neoantigen was further characterized at different levels. First, the response to Pep-2 was compared with that of its wild-type (WT) counterpart in PBMCs collected one year after vaccination. As shown in **Figure 4**, the mutated peptide elicited a stronger response compared to the WT version, evidenced by higher IFNγ secretion and increased activation of CD137 and PD-1 (**Figures 4A, B**). Based on these results, both in the post 1yr vaccination PBMCs and at the pool level after the sixth dose (Post 6), the immune response to the pool, Pep-2, and the WT version was also assessed at an earlier time point, using PBMCs collected after the third vaccine dose (Post 3). The results confirmed the secretion of IFNγ and TNF-α, both for the pool and Pep-2 at this early time point and one-year post-vaccination (**Figure 4C**). Moreover, a higher percentage of CD8+ TNF-α+ T cells was observed in PBMCs from Post 3 sample (**Figure 4D**). Together, these data point to an immune response to neoantigens that is distinct from those included in the vaccine, raising the possibility of epitope spreading—a phenomenon in which the immune system broadens its reactivity beyond the original vaccine targets, thereby enhancing the overall immune response. Nonetheless, additional assays using pre-vaccination samples would be necessary to confirm this.

To evaluate the response to this neoantigen in pre-vaccination cells, only PBMCs collected after NAC were available for analysis. Simultaneously, immunogenicity assays for Pep-2 were conducted on post-chemotherapy and one-year post-vaccination cells (Post 1yr). The results showed a CD4+ T-cell response, characterized by CD154 expression and elevated IFNγ levels in the culture supernatant (**Figures 4E, F**). However, after vaccination, the neoantigen-specific response appeared to shift toward a CD8-mediated response, as indicated by an increased frequency of CD8+CD137+ T cells and higher IFNγ levels in the supernatant following Pep-2 stimulation (**Figures 4G, H**). Notably, a response to the wild-type peptide was also observed, although at a lower magnitude. Consistently, unsupervised analysis using Phenograph identified population 20 (pink, **Figures 4 I, J**), characterized by the expression of CD8, CD137, CD154, and PD-1. This population expanded after *in-vitro* stimulation with Pep-2, primarily in PBMCs collected one-year post-vaccination, corroborating the findings from the manual analysis (**Figures 4I, J**, **Supplementary Table 2**).

These findings indicate that a response to Pep-2 was already present before vaccination; however, with the available experimental units, it is not possible to determine whether this response was caused by NAC or if it pre-existed both interventions. Since responses were observed from the time of NAC, they may instead be linked to immunogenic cell death induced by the neoadjuvant chemotherapy with doxorubicin given to this patient, a process previously reported in the literature (50, 51). In this context, vaccination appears to have enhanced the response to Pep-2, primarily mediated by CD8+ T cells that persisted for up to one year after vaccination. Nonetheless, it cannot be confirmed that epitope spreading is truly occurring, as only immunogenicity tests performed with pre-treatment cells—before both NAC and vaccination—would confirm this phenomenon.

### The implemented germinal pipeline allows for the identification of variants in cancer driver genes

**Figure 5:**
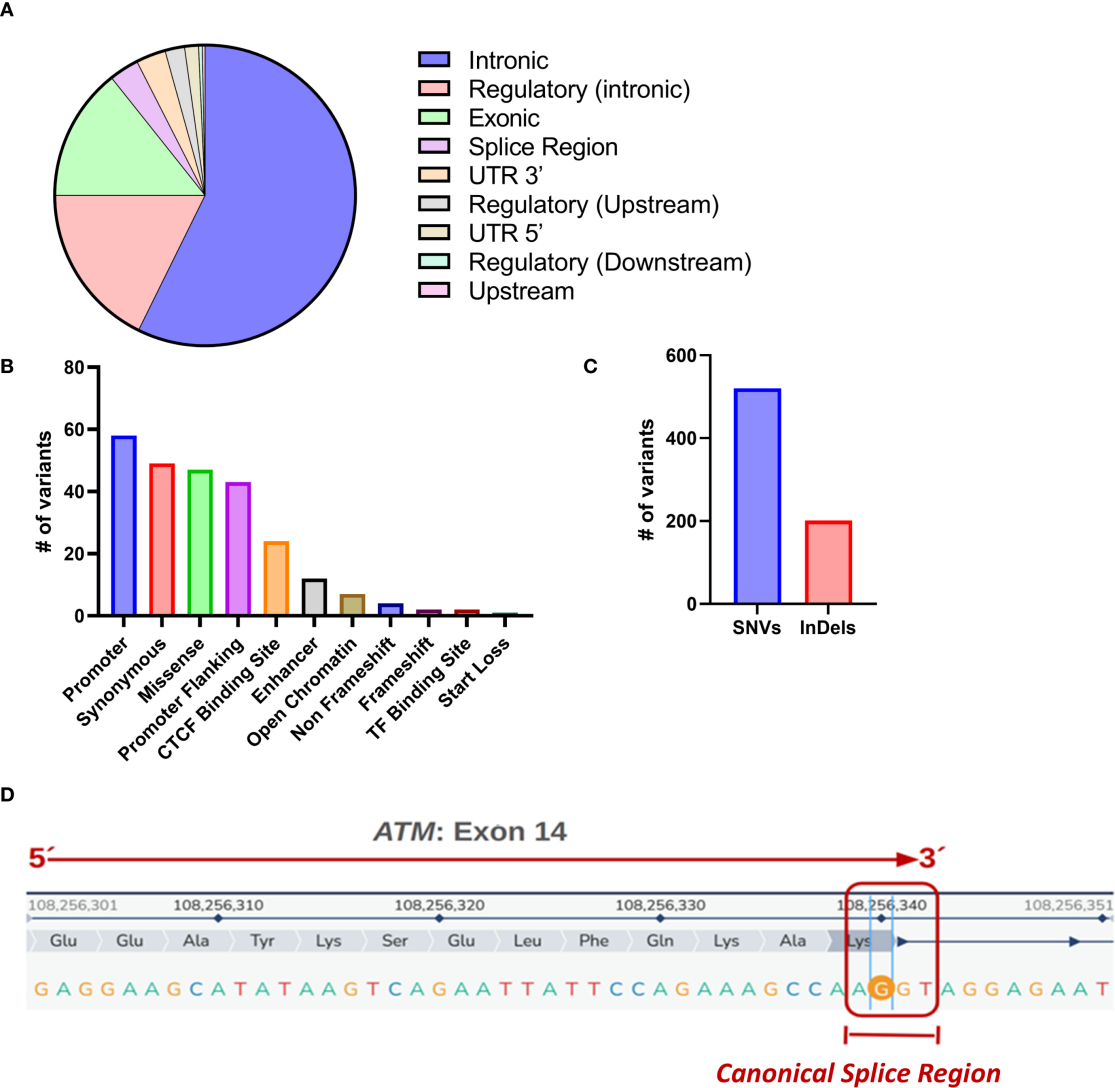
Analysis of the genomic region of all variants found in cancer driver genes. **(A)** The analysis shows that 57.3% of the variants are located in intronic regions, 17.8% are found in intronic regulatory regions, and 14.3% affect exons. Variants in splicing regions (3.2%) and in the 3’ and 5’ UTRs (3.2% and 1.5%) may influence RNA maturation and translation. The remaining variants are distributed across upstream and downstream regulatory regions (2.1% and 0.4%) and upstream variants (0.3%). These results indicate that most germline variants occur in non-coding regions of the genome. The fact that the majority of the variants are benign or probably benign (557/721) in the cancer driver genes suggests that most of them are polymorphisms not directly involved in carcinogenesis. **(B)** Number and effect of alterations in the driver genes. **(C)** Total number of variations in driver genes. SNV: single nucleotide variant. Indel: insertion/deletion. **(D)** Diagram of the alteration affecting the canonical splicing region in exon 14 of the ATM gene, categorized as pathogenic following the recommendations of the American College of Medical Genetics and Genomics (ACMG).

Given that information from the whole exome sequencing (WES) obtained from patients’ PBMCs, together with WES information from the tumor, is required for identifying the universe of tumor mutations (tumor variants herein named neoantigens), and following the best practices of the Broad Institute, we set out to develop a pipeline for identifying germline variants that was validated with results from a set of samples previously characterized by a pipeline used in clinical diagnostic practice. The WES from the patient’s PBMCs was uploaded to the pipeline and analyzed for all germline variants found in a panel comprising 686 cancer driver genes (**Supplementary Table 3**). A total of 721 variants were identified in 316 driver genes, of which 520 corresponded to single nucleotide variants (SNPs) and 201 to insertions/deletions (InDels) (**Figures 5A, B**). The analysis of the distribution of variants based on their functional effects revealed that the highest proportion was concentrated in promoter regions (58), followed by synonymous variants (49) and missense variants (47). This suggests that these variants may affect both transcriptional regulation and protein structure. Variants in promoter flanking regions (43) and CTCF binding sites (24) indicate a potential impact on regulation at the epigenetic level. Additionally, variants in enhancers (12) and open chromatin regions (7) were identified, reflecting their possible influence on chromatin accessibility. The non-frameshift (4) and frameshift (2) variants could impact the integrity of the protein reading frame, while the start loss (1) implies the loss of the start codon. The remaining variants are concentrated in deep intronic regions, making the functional effect unclear. These results underscore the importance of investigating both coding variants and those in regulatory regions of the genome.

Among the variants identified, 557 are benign or probably benign, 163 have uncertain clinical significance, and one pathogenic variant in the ATM gene affects exon 14 at c.2250G>A (NM_000051.4). Although this is a synonymous variant (p. Lys750=), it occurs at the last nucleotide of exon 14, which encodes lysine. This nucleotide position is highly conserved, and in silico prediction tools support its potential pathogenicity: SpliceAI indicates a strong splice-altering effect (0.94), while dbscSNV Ada and RF classify it as deleterious. The RF algorithm predicts that SNVs within the consensus splice regions (−3 to +8 at the 5’ splice site and −12 to +2 at the 3’ splice site) may disrupt splicing, assigning scores from 0 to 1—higher scores suggest a greater risk. Functional studies have shown that splice site substitutions can cause single exon skipping; specifically, the 2250G>A variant has been documented to result in exon skipping and loss of ATM function (52). Additionally, analysis of the patient’s tumor RNA-Seq data confirms heterozygous expression of this variant at the mRNA level, supporting its potential role as an oncogenic driver in this case.

ATM is a key kinase in the DNA damage response, particularly in repairing double-strand breaks and playing a crucial role in cell cycle checkpoint signaling (53). ATM mutations are associated with an increased risk of breast, pancreatic, and lymphoma cancers due to loss of function, which leads to the accumulation of mutations (54). Additionally, ATM serves as a significant target for combination therapies, such as radiation therapy, because of its role in maintaining genomic stability (55).

### High-confidence somatic variant detection supports tumor molecular profiling and personalized clinical decisions

We use SomaticSeq’s machine learning algorithm, which applies three confidence filters: PASS (P ≥ 0.7) for high-confidence mutations, LowQual (0.1 < P < 0.7) for ambiguous variants, and REJECT (P ≤ 0.1) for potential false positives. After filtering out and discarding the rejected variants, which are more likely to be false positives, we identified 373 affected genes and a total of 385 variants. These genes were then filtered based on cancer driver genes, and we proceeded to annotate them using the OncoKB database (https://www.oncokb.org/). The analysis found mutations in 18 cancer driver genes, of which 3 met clear oncogenicity criteria in the TP53, NF1, and PIK3CA genes with OncoKB annotation (**Supplementary Table 4**).

In our patient, high-confidence somatic mutations were identified in TP53 (p.Gly199Ter), NF1 (p.Phe1593SerfsTer31), and PIK3CA (p.Glu542Lys). The nonsense mutation in TP53 indicates a loss of function of the tumor suppressor gene, which may contribute to uncontrolled cell proliferation. The frameshift mutation in NF1 suggests inactivation of neurofibromin, leading to sustained activation of the RAS/MAPK pathway. The activating mutation in PIK3CA results in the continuous activation of the PI3K/AKT/mTOR pathway, promoting cell survival and growth (56, 57).

According to OncoKB, evidence levels are specific to indication, biomarker, and line of therapy and are presented here as contextual rather than prescriptive. In our case, the NF1 annotation (Level 1 for selumetinib; Level 4 for trametinib/cobimetinib) relates to NF1-associated plexiform neurofibromas and is not directly applicable to the tumor type presented. For PIK3CA p.E542K, OncoKB assigns Level 1 evidence for alpelisib plus fulvestrant and for capivasertib plus fulvestrant in HR+/HER2− disease; additional regimens such as inavolisib with palbociclib and fulvestrant (Level 3A) and RLY-2608 with or without fulvestrant (Level 4) are investigational. We include these annotations to provide context for potential therapeutic options if the clinical criteria are met; no treatment decision was made in this patient based solely on these findings (41, 58, 59).

## Discussion

Personalized medicine offers the opportunity to develop therapies tailored for each patient. This case report outlines an approach that utilizes genetic data from healthy and tumor tissues, resulting in treatment with dendritic cells pulsed with a predicted neoantigen, and assists in identifying clinical variants as potential therapeutic targets.

Initially, by analyzing the exome and transcriptome of the tumor cells along with the exome of healthy tissue, we could identify the patient’s tumor neoantigens and select them for vaccination. Since neoantigens are unique to tumors compared to normal tissues and are not affected by central tolerance, they serve as promising targets for generating and enhancing immune responses. Based on this, we assessed the potential of a personalized vaccine using autologous dendritic cells pulsed with neoantigens, combined with NAC, to trigger an immune response in a patient with TNBC.

In this case, vaccination was carried out by pulsing autologous DCs with a single long synthetic peptide encoding a predicted neoantigen. By analyzing PBMCs collected before and after vaccination, it was possible to investigate the immune responses triggered by this therapy. The results indicate that the neoantigen used in the vaccination is immunogenic, as shown by cytokine secretion such as IFNγ when PBMCs are stimulated with the peptide. This response increased with successive doses, evidenced by higher IFNγ secretion and greater expression of activation markers in PBMCs after the third and sixth vaccinations. Consistently, analysis of TCR-CDR3 frequency revealed shifts in the repertoire after chemotherapy that continued to expand with vaccination. Moreover, we identified TCR-CDR3 clonotypes that may be specific to the neoantigen, based on their increased frequency upon peptide stimulation. However, these findings are based on indirect evidence, and a tetramer was not available to definitively confirm the specificity of these clonotypes. Therefore, additional functional assays will be necessary to determine whether specific TCR sequences directly mediate recognition of the neoantigen.

Furthermore, we assessed the immunogenicity of other neoantigens not included in the vaccine. Stimulating PBMCs with these peptides showed an immune response to one of the four neoantigens tested, and this response was already detectable in NAC sample but pre-vaccination cells. Notably, a change in the pattern of responding cells was observed: before vaccination, the response was mainly mediated by CD4+ T cells, whereas after vaccination, it was primarily driven by CD8+ T cells.

These findings align with the concept of epitope spreading, which has been observed in previous neoantigen vaccine studies, where vaccine-induced T cells kill tumor cells and promote the release of more antigens that in turn trigger new T-cell responses (16, 60–63). However, our results also showed that Pep-2–specific responses were already detectable in the NAC sample, suggesting this phenomenon cannot be solely attributed to vaccination. Instead, immune priming may have been triggered by immunogenic cell death caused by doxorubicin-based neoadjuvant chemotherapy, a mechanism known from previous research (50, 64). In this context, our data imply that NAC may have primed Pep-2–specific T cells, and vaccination then boosted this response, as seen by their persistence and expansion up to one year after vaccination. Nevertheless, the lack of pre-intervention sample (before) when evaluating this second set of neoantigens prevents us from knowing whether these responses were already present before treatment. This limitation restricts our ability to draw firm conclusions about epitope spreading and emphasizes the need for functional assays at earlier time points.

Ultimately, our study demonstrated that personalized neoantigen-based vaccines could be a promising immunotherapy option for TNBC patients with persistent tumors after NAC. Vaccination with DCs loaded with neoantigens elicited an *in vitro* immune response that strengthened with each vaccination cycle and may help reduce the risk of relapse. However, long-term patient monitoring will be essential to evaluate the durability and clinical effectiveness of this approach.

Secondly, the examination of the exome in both tumor tissue and healthy tissue helped identify germline and somatic variants that are relevant for therapy. On one side, the germline variant ATM:c.2250G>A (p.Lys750=) was found in our patient, which has been reported in people with hereditary cancer. This variant has 28 entries in the ClinVar database, all of which are classified as pathogenic or probably pathogenic (Accession: VCV000003044.76). A case in the literature (65) describes a 49-year-old patient diagnosed with breast cancer who has this same variant. Expert panels such as ClinGen and Decipher recognize the link between pathogenic variants in the ATM gene and hereditary breast and ovarian cancers, supported by publicly available resources (Decipher, ClinGen). All this information highlights the clinical importance of pathogenic or probably pathogenic variants in the germline of patients, relating to their risk for hereditary cancer. The pipeline established here follows GATK best practices and offers a strong method to identify and annotate germline variants in cancer patients.

The bioinformatic approach in this work combines multiple somatic variant detection tools by using various callers and incorporating machine learning to accurately identify tumor mutations. The pipeline integrates tools like MuTect2, MuSe2, Strelka2, LoFreq, and Somaticseq to enhance sensitivity and specificity through an integrated analysis that generates a consensus of mutations. SomaticSeq employs a supervised machine learning model to prioritize somatic variants and reduce false positives by extracting features from sequencing reads, thereby increasing the reliability of tumor-specific mutations. Tests with cancer data show that SomaticSeq outperforms individual tools, especially in managing tumor heterogeneity and sequencing artifacts. Combining traditional methods with machine learning models enables more confident detection of somatic mutations, which is crucial for genomic studies in oncology.

Our somatic pipeline includes strict QC, consensus calling, and clinically focused annotation (OncoKB) to interpret the biological effects and therapeutic relevance—specific to indication and treatment line—of genomic alterations. In this study, these annotations provide hypothesis-generating evidence—linked to tumor type, biomarker, and regulatory context—rather than serving as direct treatment recommendations. No treatment decisions for this patient were made solely based on these findings. Our bioinformatics approach has enabled us to identify targeted therapies through genomic profiling of the patient’s tumor. We believe our platform is based on a robust and reproducible method for identifying somatic variants with clinical significance, which may support future clinical decision-making when combined with appropriate clinical and regulatory criteria.

In conclusion, this case report demonstrates the feasibility and potential of personalized neoantigen vaccination combined with NAC for TNBC. The study shows that integrated genomic profiling can identify immunogenic neoantigens capable of eliciting specific T-cell responses, as well as clinically actionable germline and somatic variants that may inform tailored therapeutic strategies. Although limited to a single case, these findings emphasize the importance of utilizing precision oncology approaches to improve patient outcomes and support further investigation of this strategy in larger clinical trials for effectiveness and broader use.

## Data Availability

The data analysed in this study were obtained from the Immunology and Translational Medicine Research Group (GI&MT) of the Universidad Nacional de Colombia, http://investigacionimt.unal.edu.co. The following licences/restrictions apply: sharing of this dataset will occur only after approval by the Medical School Ethical Review Board and formalization of a Data Transfer Agreement (DTA). Requests to access these datasets should be directed to Professor Carlos Alberto Parra-López, MD, Ph.D., Principal Investigator of this study (caparral@unal.edu.co; grupoimtfm@bog@unal.edu.co).
